# Food Security, Financial Resources, and Mental Health: Evidence during the COVID-19 Pandemic

**DOI:** 10.3390/nu14010161

**Published:** 2021-12-30

**Authors:** Jackie Yenerall, Kimberly Jensen

**Affiliations:** Department of Agricultural and Resource Economics, University of Tennessee, 302 Morgan Hall, 2621 Morgan Circle, Knoxville, TN 37996, USA; kjensen@utk.edu

**Keywords:** food insecurity, mental health, financial resources, COVID-19

## Abstract

COVID-19 has negatively impacted many households’ financial well-being, food security, and mental health status. This paper investigates the role financial resources play in understanding the relationship between food security and mental health among U.S. households using data from a survey in June 2020. Results show job loss and savings draw down to pay for household bills had a significant relationship with both lower food security and greater numbers of poor mental health days during the pandemic.

## 1. Introduction

Food insecurity occurs when households lack sufficient resources to access enough food to maintain a healthy life [1]. Addressing household food insecurity is of interest to policymakers in part because prior research has shown it has a negative effect on physical health [2]. More recently researchers have found evidence to suggest there may also be a relationship between food insecurity and poor mental health [3,4]. One of the challenges in isolating the influence of food insecurity on mental health is addressing the influence that financial resources have on both food insecurity and mental health. This relationship is particularly concerning during the COVID-19 pandemic, which has had implications not only for household’s physical and mental health but also their financial well-being. The purpose of this article is to investigate the role financial resources play in understanding the relationship between food security and mental health using data collected in the United States (U.S.) in June 2020.

While food security refers specifically to a household’s ability to access sufficient food, financial resources are used to meet households’ basic needs more broadly. Financial resources can include income, employment, participating in government safety net programs, savings, and ownership of assets like homes. The importance of financial resources in determining food security in the U.S. is evident in the structure of the survey questions used by the federal government to assess food security prevalence annually in the Food Security Supplement, which is part of the Current Population Survey (CPS) [1]. Each item in the survey qualifies that the food related behavior or condition under question occurred due to lack of money or issues related to affordability of food [1].

However, past research has found income alone is insufficient to predict food security status and other indicators of financial resources play an important role [5,6]. In particular, renting instead of owning a home, inadequate savings or having to use savings to pay for bills, and loss of employment or income are all associated with an increased likelihood of household food insecurity [7,8,9,10]. Prior research has also found, after controlling for selection bias resulting from the program participation decision, that participation in the Supplemental Nutrition Assistance Program (SNAP) decreases the likelihood of food insecurity [6].

Prior research investigating the relationship between financial resources and mental health has also found that job or income loss and living through a recession are associated with poor mental health outcomes, such as increased levels of depression or anxiety [11]. However, there is generally limited consideration for the influence of financial resources when examining the relationship between food security and mental health. Prior research tends to only control for income level or employment status [12,13,14,15,16,17], and only a few studies control for additional measures of financial resources, such as job or income loss, and participation in government safety net programs [18,19].

Given the influence of financial resources on food security and mental health independently, it suggests that it is important to adequately control for financial resources when estimating the relationship between food security and mental health [5,6,11]. Failure to control for the influence of financial resources beyond income could bias the estimated relationship between food security and mental health status. Addressing the relationship between food security, financial resources, and mental health is particularly important during the COVID-19 pandemic, as the pandemic and subsequent recession, which lasted from March to April 2020, have had significant implications for both the health and economic well-being of households in the U.S. [9,20,21].

In late March 2020, state governments began ordering business closures to mitigate the spread of COVID-19 and households began limiting their in-person activities to reduce their exposure to the virus [22]. The result was a historic rise in unemployment rates, particularly amongst low-income and low education households who were previously employed in sectors that could not transition to work from home formats [9]. Job losses due to COVID-19 related business closures and loss of income during this time period contributed to a rise in food insecurity and insufficiency [9,23]. By May of 2020 the prevalence of household food insecurity had reached 15.4%, a level similar to that during the Great Recession [23]. The prevalence of food insufficiency, which occurs when households sometimes or often do not have enough to eat, increased from 3.4% in 2019 to 10.8% in April 2020 based on Census data, exceeding the peak during the Great Recession [20].

The negative effect of the COVID-19 pandemic on employment, income, and food security would also be expected to influence mental health status. We are aware of three papers that have previously assessed the influence of food insecurity on mental health during the COVID-19 pandemic [17,18,19]. While all three found that food insecurity or insufficiency was associated with a statistically significant increase in the likelihood of anxiety, depression, or stress [17,18,19] only Fang et al. (2021) [18] included both measure of how employment and income were impacted by the COVID-19 pandemic, as well as participation in several government safety net programs. However, Fang et al. (2021) [18] only used a binary indicator of food insecurity. This specification of the food security variable limited their ability to investigate a potential gradient in the influence of food security status on mental health that has been identified in previous literature, including during the pandemic [12,16,17].

The purpose of this article is to further investigate the role of financial resources in understanding the relationship between food security and mental health during the COVID-19 pandemic. To accomplish this, we estimate two models. First, we estimate the influence of financial resources on food security. Second, we estimate the influence of food security and financial resources on mental health. We not only include the more commonly used measures of financial resources such as income, but also additional measures of financial resources such as use of savings, changes in income or employment, use of charitable food, and participation in government programs [5,6]. Should the inclusion of additional measure of financial resource substantially modify the relationship between food security and mental health status it would suggest their absence could result in an omitted variable bias.

Identifying potential sources of omitted variable bias is important for future research as it highlights the need for additional data collection or methodological considerations when investigating the relationship between food security and mental health. It is also important for policy makers as it suggests a potential mechanism from changes in financial resources to both food security and mental health. While traditional food security programs and policies focus on improving access to food, if financial resources influences both food security and mental health it may suggest policies designed to improve financial resources will benefit both food security and mental health. It is important to note that in addition to the potential omitted variable bias discussed above, there is also the potential for reverse causality in the relationship between food insecurity and mental health status. Thus, the results of our study cannot be used to infer a causal relationship between food insecurity and mental health.

Additionally, in our analysis we measure food security using the different levels (e.g., food secure, marginally food secure, low food security, and very low food security) to detect any potential gradients in the relationship between food security, financial resource, and mental health. The levels of food security status capture the continuum of the food insecurity condition, which becomes more severe as household move from marginal food security to very low food security [1]. In particular, low levels of food security suggests households have had to modify their food purchases or consumption for reasons related to financial resource constraints [1]. Thus, these households may not only be the most sensitive to changes in financial resources, but also in greatest need of assistance. Understanding the relationship between levels of food security and financial resources and mental health could help to develop policies that better target those households in greatest need.

## 2. Materials and Methods

### 2.1. Data and Measurement

The data for this study came from a national convenience sample of 2000 respondents collected using an online survey administered by Qualtrics in July 2020. Respondents were included if they were at least 18 years of age, the household’s primary food shopper, lived in the U.S. and had lived in the same state since 1 February 2020. Quotas were included in the sampling procedure to reflect the distribution of U.S. households according to the American Community Survey (ACS) based on their 2019 income (<USD 25,000, USD 25,000 to USD 49,999, USD 50,000 to USD 74,999, USD 100,000 plus), age (18–34 years, 35–54 years, 55 years and older) and geographical region (i.e., Northeast, Midwest, West and South). The survey was pre-tested using 50 respondents before being released for broader distribution. Appropriate human subjects’ protocols were followed, and institutional review board approvals were obtained (UTK-IRB-20-05882-XM).

The survey collected information on respondent demographics and mental health status in the past thirty days as well as measures of household characteristics, socioeconomic status, and food security status in the past thirty days. Thus, the survey provides measures of respondent’s mental health status and household food security status for June 2020. Descriptions of how the food security, mental health, and financial status variables were formulated is provided in the following paragraphs.

Household food security status in the past thirty days was measured using the USDA-ERS six-item short form version of the USDA-ERS U.S. Adult Food Security Module [23]. Based on the number of affirmative responses to questions regarding their ability to meet basic food needs due to limited resources households were classified into one of four food security categories: high food security (0 affirmative responses), marginal food security (1 affirmative response), low food security (2 to 4 affirmative responses), and very low food security (5 to 6 affirmative responses) [1,24].

Respondent’s mental health in the past thirty days was assessed by asking “how many days would you say you have experienced greater than usual levels of problems with stress, depression, or other emotional issues?” This question was adapted from the Behavioral Risk Factor Surveillance Systems (BRFSS) question for the mental health portion of the Healthy Days Measures [25]. Responses included none, 1 to 7 days, 8 to 14 days, 15 to 21 days, and 22 to 30 days. Responses to the question create a polychotomous ordinal measurement of general mental health status, where higher values indicate more days of poor mental health.

Households were asked a series of questions to assess their current level of financial resources. An indicator for whether a household was below 130% of the Federal Poverty Line (FPL) poverty line was constructed based on the household’s annual income in 2019 and the household size. Households were also asked if the owned or rented their current home, if they used money from savings to pay for household bills in June or had no savings available in June, used of charitable food from any source in June, and about their current participation in government safety net programs.

Sources of charitable food listed in the survey included food bank or food pantries, shelter or soup kitchen, family or friends or neighbors purchased groceries or prepared a meal for the household, and religious organizations. Participation in government safety net programs included: unemployment insurance, Woman Infants and Children (WIC), and Supplemental Nutrition Assistance Program (SNAP). We also asked if they had received benefits from two COVID-19 pandemic specific programs: coronavirus stimulus checks and Pandemic Electronic Benefits (P-EBT). The latter was a program designed to replace the value of free and reduced-price lunches lost due to school closures.

Finally, to capture recent changes in financial resources related to the pandemic, binary indicators were created for respondents that had lost their job during the COVID-19 pandemic or had experience a change monthly income in June 2020 as compared to January 2020.

Respondent characteristics included age, education level, and self-reported race, ethnicity, and gender. Household characteristics included an indicator for the presence of at least one child in the household, the Census region in which the household lives, and an indicator for if the household lives in a nonmetropolitan county. Survey respondents were asked to report the county and state in which they reside, and that information was used to determine the household’s Census region (i.e., Northeast, South, Midwest, West) and nonmetropolitan status based on the United States Department of Agriculture (USDA) Economic Research Service’s (ERS) 2013 Rural Urban Continuum Codes (RUCC) [26].

### 2.2. Statistical Analysis

Although the sampling procedure implemented by Qualtrics included quotas to capture a nationally representative sample, online surveys often fall short of being truly representative due to limitations in sample sizes and sampling procedures [27]. To address sample imbalance, we used iterative proportion fitting, also known as raking, to create probability weights based on gender, age, household size, race, income, and educational status using controls from the 2019 American Community Survey (ACS) [27,28]. All respondents with complete responses to these variables were used in the raking analysis (*n* = 1936). After the probability weights were created respondents without complete responses to the variables used in the regression analysis were dropped to create the final analytical sample (*n* = 1567).

The resulting probability weights were included in our descriptive and regression analysis. Weighted descriptive statistics were calculated to compare across households based on their food security status, and a weighted f-statistic was calculated to test for differences across food security status.

A weighted multinomial logistic regression (MNL) was used to investigate the relationship between financial resources and the different levels of food security (i.e., high, marginal, low and very low foods security). Average marginal effects (AME) were reported and can be interpreted as the change in the likelihood of observing a particular food security status for a change in a given covariate. All financial resource variables were included with the exception of participating in government safety net programs, due to previous research demonstrating a selection bias associated with the choice to participate in programs like SNAP and food security status [6].

Since days of poor mental health were recorded in one-week increments, rather than a continuous measure of days in the past month, a weighted ordered proportional logistic regression was used to estimate the influence of financial resources and food security status and odds ratios (OR) were reported. Four separate models were estimated to better understand the influence of financial resources on the relationship between food security status and mental health. Three models included the food security indicators, and a fourth did not include the food security indicators in order to estimate the direct influence of the financial resource variables on mental health status.

Within the three models that included the food security indicators the first model included only one measure of financial resources, an indicator that household income was below 130% FPL, to be consistent with previous research that most commonly only includes an indicator of income [12,14,15,16,17,29]. The second model includes additional measures of financial resources including the indicator for renting a home, losing a job during pandemic, changes in monthly income, use of savings, utilizing charitable food sources, and receiving a coronavirus stimulus check. Finally, participating in government programs including WIC, SNAP, and unemployment were added to the model. These were included last due to the potential to introduce sample selection bias into the model.

The same set of respondent and household characteristics were used in all regressions and included age, indicators for identifying as black, white, or Hispanic, the presence of children in the home, less than a college degree, census region indicators, and an indicator for resident in a non-metropolitan county.

Only respondents with complete responses for the variables utilized in the analysis were include, which resulted in an analytical sample size of 1567 respondents. All analysis was conducted in Stata version 16.

## 3. Results

### 3.1. Descriptive Statistics

Within our sample 52.79% of households had high food security, 10.65% were marginally food secure, 20.50% were low food secure, and 16.06% were very low food secure. Table 1 and Table 2 contain weighted descriptive statistics to compare household characteristics and financial resources, respectively across the four levels of food security: high food security, marginal food security, low food security, and very low food security. A weighted F-test was used to first test for a difference across all four food security categories.

Table 1 demonstrates a gradient across food security status for several respondent and household characteristics, including age, presence of a child, and education. The average age of respondents decreases with the level of food security. Respondents in high food security households are oldest with an average age of 54.04 years while the average age of respondents from low and very low food security households are similar at 38.13 and 39.07 years, respectively. Perhaps related, the proportion of households with a child present increases across the food security gradient from 22.23% in high food security households to 53.73% in very low food security household.

Consistent with previous research, food secure households in our sample are more likely to be white and less likely to have a high school degree or less [5]. Amongst high food security households, 71.86% of respondents are white while only 5.33% are black. Compared to the prevalence of white and black respondents in the overall sample, 63.95% and 10.85%, respectively, this demonstrates that black respondents are disproportionately less likely to be in high food security households. On the other hand, amongst very low food security households 56.70% of respondents are white, while 16.34% are black. The prevalence of respondents with a high school degree or less also increases from a low of 28.97% amongst high food security households, to around 44% for low or very low food security households.

Table 1 also contains descriptive statistics for the mental health outcome. Since the variable is ordinal, we report the percentage of households that report any days of poor mental health in the past month, or two weeks or more of poor mental health in the past month. In both cases, there is a strong and clear increase in the prevalence of experiencing more days of poor mental health when comparing high food security to very low food security households. While 55.38% of high food security household report having at least one day of poor mental this increases to 67.13% for marginally food secure households, to 78.12% for low food security households, and 88.09% for very low food security households, Similarly, the prevalence of respondents reporting at least two weeks of poor mental health increases from a low of 12.15% in high food security households, to 20.90% of marginally food secure households, to 24.15% of low food security households, to a high of 35.75% amongst very low food security households.

Table 2 reports descriptive statistics for the financial resource variables. The table shows that there is a statistically significant difference for nearly every financial resource variable, with a gradient across food security categories almost always indicating access to fewer financial resources amongst household with lower levels of food security. Considering household’s 2019 income to FPL ratio, only 3.31% of high food security households had an income below 100% FPL, compared to 18.42% and 14.02% of low and very low food security households. Mirroring this pattern, 81.58% of high food security households had incomes exceeding 185% FPL, compared to 57.18 and 59.26% of low and very low food security households.

The percentage of households who had experienced a disruption to their household’s finances was also higher amongst households who were less food secure. Only 1.79% of respondents in high food security households had lost their job, compared to 9.09% of respondents in very low food security households. Similarly, 16.97% of high food security households reported that their monthly income had decreased during the pandemic as compared to 45.55% of very low food security households.

The need to use savings to pay for bills or the use of charitable food sources was also the highest amongst the least food secure households. While 27.67% of high food security household reported needing to use savings, this increased to 48.69% amongst marginally food secure households, 58.43% for low food security households and 60.92% for very low food security households. Additionally, approximately 29.78% of very low food security household reported having no available savings in June, as compared in 6.88% of high food security households. Finally, for each of the federal safety net programs, the prevalence of households participating increases across all programs with the exception of WIC, in which low food security households have a higher prevalence of participating homes than the very low food security households.

### 3.2. Regression Results

Since the multiple measures of financial resources could be correlated when used in the regressions with either food security or days of poor mental health in the past month as the dependent variable, we utilized the variance inflation factors (VIF) to assess multicollinearity. The average VIF for all models are reported in the footnotes of Table 3 and Table 4. A VIF under 10 would suggest that multicollinearity is not problematic and as the results show the average VIF for all models falls below this threshold [29].

Table 3 contains the estimated average marginal effects (AME) from the weighted multinomial logistic regression for food security status and financial resources. In general, the results show that a lack of financial resources increased the probability of being less food secure. Having an annual income of less than 130% FPL decreased the probability of having high food security (AME = −0.13) but increased the probability of low food security (AME = 0.12). Experiencing a decrease in monthly income decreased the probability of having high food security (AME = −0.08) but increased the probability of very low food security (AME = 0.12). On the other hand, an increase in monthly income decreased the probability of low food security (AME = −0.10) but increased the probability of very low food security (AME = 0.13).

The variables capturing the use of savings to pay for bills in June were amongst the strongest predictors of all the financial resource variables. Needing to use savings decreased the likelihood of having high food security (AME = −0.23) but increased the probability of either low (AME = 0.09) or very low food security (AME = 0.13). Similarly, not having any savings available decreased the probability of having high food security (AME = −0.27) but increase the probability of either low (AME = 0.07) or very low food security (AME = 0.23). The use of charitable food had a similar influence. Receiving charitable food from any source decreased the probability of having high food security (AME = −0.18) but increased the probability of low (AME = 0.08) and very low food security (AME = 0.18).

Table 4 contains the proportional odds ratios from the ordered logistic regression for the mental health outcome. The first three models included the financial resource and food security category variables, while the fourth model contains the financial resource variables only. Model 1 in Table 4 is most consistent with past research in that it only includes demographic variables and a single measure of financial resources, an indicator for household income below 130% FPL. Similar to previous research, we find that lower food security has a strong influence on the odds of experiencing more days of poor mental health. Relative to having high food security, the odds of more days of poor mental health for marginal food security were 1.85 times greater, or 2.19 times greater for low food security, and 3.74 times greater for very low food security.

Model 2 in Table 4 adds the more expansive measures of financial resources, with the exception of variables capturing participation in government safety net programs. Again, we find that decreasing food security has a strong influence on the odds of experiencing more days of poor mental health, however, the magnitude of the relationship is attenuated by the additional controls for financial resources. Specially, the proportional odds ratio for marginal food security, low food security, and very low food security are 17%, 18%, and 29% smaller as compared to the proportional odds ratio from Model 1.

Of the included additional financial resource variables, only experiencing a decline in monthly income and using savings had a statistically significant impact on the proportional odds for increasing days of poor mental health. The odds of experiencing more days of poor mental health are 1.65 times greater for households that experienced a decline in monthly income, and 1.42 times greater for households that had to use savings to pay for bills. The results are largely unaffected by adding the variables for participating in government safety net programs in Model 3. While this was an unexpected finding it is consistent with Fang et al. (2021) [18].

Model 4 in Table 4 shows that excluding the food security category variables from the regression had minimal impact on the findings. Unlike in the previous models renting a home and receiving charitable food had a statistically significant effect on the proportional odds of poorer mental health. This model also shows that the two financial resource variables, a decrease in monthly income, and using savings, had a direct effect on days of poor mental health.

Across all four models the effect of the demographic covariates remains largely stable when considering either the statistical significance or the magnitude of the effect. Only the indicator for live in the Midwest changes in significance after adding the additional financial resources variables in the second column of Table 4.

## 4. Discussion

The COVID-19 pandemic has had a significant impact on the health and economic well-being of many households in the United Status. This study seeks to illuminate the relationship between financial resources, food security, and mental health status of households during the pandemic. Similar to previous studies, we find that decreasing levels of food security are associated with an increase in more days of poor mental health. However, we also find that including more detailed measures of financial resources substantially weakened measures of this relationship. In particular, we find that losing a job during the pandemic and having to use money from savings to pay for bills had a significant relationship with both the probability of both low and very low food security and increased the odds of greater days of poor mental health during the pandemic. This suggests that a failure to adequately control for financial resources may create an omitted variable bias when estimating the relationship between food security and mental health. Thus, future researchers may want to consider collecting more extensive data on financial resources and examining the relationship between changes in financial resources, food security, and mental health in more detail.

Even after controlling for additional financial resources, the odds of experiencing more days of poor mental health were 2.6 times greater in households with very low food security as compared to high food security households. This is particularly concerning because very low food security is categorized by a modification in food expenditures or consumption. However, households experiencing very low food security may lack the financial resources to address either their food security or mental health needs and require additional assistance. The results from this study suggest that future research may want to investigate the potential for program design to address financial resources to benefit both food security and mental health outcomes.

There are several limitations to this study. First, this study uses cross-sectional data, and the results cannot be used to infer a causal relationship. In particular, we acknowledge that there are also studies that have found that poorer mental health increases the likelihood of food insecurity, which raises the possibility of reverse causality in our study [13]. Thus, future studies need to focus additional attention on identify the potential mechanisms relating food security and mental health. Additionally, unlike several other studies, we use a general measure of mental health where the outcome captures days of poor mental health [17,18,19]. Thus, our results may not be directly comparable.

## 5. Conclusions

This analysis has demonstrated the important role that household financial resources play in understanding the relationship between food security and mental health. In particular, our study found that a decline in monthly income and the use of savings to pay for bills had a direct association with both food security status and mental health outcomes. Specifically, the use of savings to pay for bills increased the likelihood of either low or very low food security and increased the odds of more days or poor mental health in the past month. While a decline in monthly income increased the likelihood of very low food security and the odds of more days or poor mental health in the past month.

Furthermore, our results demonstrate that failing to control for more detailed measures of financial resources, including changes in monthly income or the use of savings to pay for bills, results in an overestimation of the relationship between food insecurity and mental health. The proportional odds ratio for marginal food security, low food security, and very low food security were 17%, 18%, and 29% smaller after controlling for more detailed measures of financial resources. Thus, future researchers will need to address the influence of household financial resource when trying to isolate the relationship between food insecurity and mental health.

## Figures and Tables

**Table 1:** Respondent and household weighted characteristics in June 2020.

	Full Analytical Sample	High Food Security	Marginal Food Security	Low Food Security	Very Low Food Security	F-Test
Weighted Mean (Linearized SD)	N = 1567	N = 800	N = 173	N = 325	N = 269	
Female (%)	55.79 (1.83)	58.80 (2.43)	65.78 (4.81)	48.84 (3.85)	48.14 (5.08)	*
Age	47.70 (0.68)	54.04 (0.80)	47.73 (1.72)	38.13 (1.17)	39.07 (1.62)	***
Presence of children (%)	32.09 (1.67)	22.23 (1.84)	38.85 (4.52)	37.01 (3.54)	53.73 (5.04)	***
Self-Reported Race and Ethnicity (%)						
White	63.95 (1.89)	71.86 (2.40)	58.45 (5.01)	52.12 (3.93)	56.70 (5.60)	***
Black	10.85 (1.42)	5.33 (1.04)	18.92 (4.36)	16.54 (3.26)	16.34 (5.89)	***
Multiple or other race	8.82 (0.95)	9.02 (1.29)	7.93 (2.87)	8.59 (2.09)	9.06 (2.53)	
Hispanic	16.37 (1.66)	13.79 (2.22)	14.70 (3.79)	22.67 (3.89)	17.90 (4.69)	
Education (%)						
Highschool or less	35.50 (1.89)	28.97 (2.48)	39.06 (4.94)	44.26 (3.98)	43.43 (5.49)	**
Some college or 2-year degree	31.24 (1.54)	34.27 (2.17)	31.47 (4.52)	26.55 (3.25)	27.11 (3.49)	
Completed college	20.92 (1.30)	23.72 (1.76)	22.43 (3.73)	16.60 (2.52)	16.27 (4.04)	
Graduate degree	12.33 (0.90)	13.04 (1.37)	7.04 (1.61)	12.59 (1.89)	13.19 (2.12)	*
Less than college education	66.74 (1.53)	63.24 (2.12)	70.53 (4.01)	70.81 (3.08)	70.54 (4.36)	
Geographic Region (%)						
Northeast	16.55 (1.24)	16.70 (1.62)	18.24 (3.47)	21.48 (3.52)	8.64 (1.76)	***
Midwest	21.40 (1.41)	24.85 (2.12)	19.59 (3.90)	19.82 (2.70)	13.27 (2.66)	**
South	37.93 (1.72)	36.78 (2.25)	37.82 (4.70)	32.80 (3.42)	48.32 (5.22)	
West	24.13 (1.60)	21.68 (2.02)	24.35 (4.37)	25.90 (3.68)	29.78 (4.88)	
Nonmetro	12.69 (1.05)	12.76 (1.47)	15.28 (3.52)	9.87 (2.01)	14.30 (2.78)	
Poor Mental Health Days in the Past Month (%)						
Any days in the past month	66.55 (1.67)	55.38 (2.37)	67.13 (4.61)	78.12 (3.23)	88.09 (2.90)	***
More than 2 weeks in the past month	19.41 (1.33)	12.15 (1.43)	20.90 (3.71)	24.51 (3.37)	35.75 (4.58)	***

* = *p* < 0.05 ** = *p* < 0.01 *** = *p* < 0.001.

**Table 2:** Household financial resource availability: weighted descriptive characteristics in June 2020.

	Full Analytical Sample	High Food Security	Marginal Food Security	Low Food Security	Very Low Food Security	F-Test
Weighted Mean (Linearized SD)	N = 1567	N = 800	N = 173	N = 325	N = 269	
2019 Income to federal poverty line (FPL) (%)						
Income ≤ 100% FPL	8.97 (0.80)	3.31 (0.62)	11.22 (2.90)	18.42 (2.62)	14.02 (2.40)	***
100% FPL < Income ≤ 130% FPL	11.49 (1.17)	8.97 (1.36)	10.25 (3.39)	17.45 (3.34)	12.99 (2.96)	
130% FPL < Income ≤ 185% FPL	7.85 (0.86)	6.14 (1.15)	9.21 (2.33)	6.96 (1.54)	13.73 (2.85)	
Income > 185% FPL	71.69 (1.52)	81.58 (1.78)	69.32 (4.45)	57.18 (3.81)	59.26 (4.70)	***
Lost job in pandemic	4.66 (0.70)	1.79 (0.45)	5.92 (2.16)	7.96 (2.20)	9.09 (2.50)	***
Self-reported change in monthly income from January 2020 to June 2020 (%)						
Income is higher	11.27 (1.40)	8.95 (1.25)	7.42 (2.67)	9.55 (2.07)	23.66 (6.21)	
Income is about the same	63.66 (1.73)	74.08 (1.99)	64.87 (4.61)	61.97 (3.66)	30.79 (4.26)	***
Income is lower	25.06 (1.43)	16.97 (1.70)	27.71 (4.27)	28.48 (3.37)	45.55 (4.96)	***
Use of savings to pay bills (%)						
Used savings	41.56 (1.78)	27.67 (2.10)	48.69 (4.88)	58.43 (3.81)	60.92 (4.69)	***
No available savings in June	13.00 (1.07)	6.88 (1.05)	10.29 (2.32)	17.01 (2.83)	29.78 (4.13)	***
No need to use savings	45.45 (1.76)	65.45 (2.21)	41.02 (4.73)	24.56 (3.38)	9.30 (2.56)	***
Housing (%)						
Rent	31.85 (1.64)	25.39 (2.08)	33.21 (4.63)	39.66 (3.94)	42.21 (4.78)	**
Own	38.14 (1.70)	39.18 (2.30)	44.47 (4.85)	35.69 (3.52)	33.67 (4.74)	
Other	30.00 (1.69)	35.43 (2.26)	22.32 (3.82)	24.65 (3.25)	24.12 (5.81)	**
Received stimulus check	76.68 (1.50)	80.31 (1.95)	80.60 (3.60)	67.86 (3.50)	73.41 (4.45)	*
Use of charitable food from any sources (%)	38.29 (1.76)	22.75 (1.93)	33.90 (4.41)	56.56 (3.90)	68.99 (4.04)	***
Participation in government programs (%)						
Supplemental Nutrition Assistance Program (SNAP)	28.50 (1.58)	12.26 (1.65)	29.87 (4.31)	50.24 (3.87)	53.20 (5.28)	***
Women Infants and Children (WIC)	14.18 (1.26)	4.42 (1.20)	13.58 (3.31)	30.89 (3.51)	25.31 (4.24)	***
Unemployment	19.11 (1.41)	11.86 (1.49)	17.82 (3.57)	29.40 (3.62)	30.69 (4.68)	***
Pandemic Electronic Benefits Transfer (P-EBT)	16.78 (1.35)	9.10 (1.31)	12.95 (3.10)	27.51 (3.68)	30.89 (4.61)	***

* = *p* < 0.05 ** = *p* < 0.01 *** = *p* < 0.001.

**Table 3:** Average marginal effects from a weighted multinomial logistic regression for the relationship between financial resources and food security status.

AME ^1^ (Delta-Method Std. Err.)	High Food Security	Marginal Food Security	Low Food Security	Very Low Food Security
2019 annual income less than 130% FPL	−0.13 *** (0.04)	0.01 (0.03)	0.12 *** (0.03)	1.41 × 10^−3^ (0.02)
Rent home	−0.03 (0.03)	2.27 × 10^−3^ (0.02)	−1.32 × 10^−3^ (0.03)	0.03 (0.02)
Lost job in pandemic	−0.12 (0.07)	0.06 (0.05)	0.05 (0.05)	0.01 (0.04)
Monthly income higher	0.02 (0.04)	−0.05 (0.04)	−0.10 * (0.04)	0.13 *** (0.03)
Monthly income lower	−0.08 * (0.03)	3.95 × 10^−3^ (0.02)	−0.04 (0.03)	0.12 *** (0.02)
Used savings	−0.23 *** (0.03)	0.01 (0.02)	0.09 ** (0.03)	0.13 *** (0.03)
No savings available	−0.27 *** (0.04)	−0.04 (0.03)	0.07 * (0.04)	0.23 *** (0.03)
Received stimulus check	0.03 (0.03)	0.02 (0.02)	−0.05 (0.03)	0.01 (0.02)
Received any charitable food	−0.18 *** (0.03)	−0.03 (0.02)	0.08 *** (0.02)	0.13 *** (0.02)
Age	4.72 × 10^−3^ *** (8.18 × 10^−4^)	6.69 × 10^−4^ (6.78 × 10^−4^)	−4.47 × 10^−3^ *** (7.47 × 10^−4^)	−9.25 × 10^−4^ (6.89 × 10^−4^)
Black	−0.15 * (0.06)	0.10 * (0.05)	0.09 (0.06)	−0.03 (0.05)
White	2.01 × 10^−3^ (0.04)	−1.34 × 10^−4^ (0.04)	0.01 (0.04)	−0.01 (0.04)
Hispanic	0.05 (0.06)	−0.01 (0.05)	0.02 (0.05)	−0.06 (0.05)
Presence of child	−0.03 (0.03)	0.05 * (0.02)	−0.07 ** (0.03)	0.05 * (0.02)
Less than a college degree	−0.04 (0.03)	0.03 (0.02)	−1.67E−03 (0.03)	0.01 (0.03)
Midwest	0.08 (0.04)	−0.03 (0.03)	−0.06 (0.04)	0.02 (0.03)
South	0.05 (0.04)	−0.03 (0.03)	−0.11 ** (0.04)	0.10 ** (0.03)
West	−0.04 (0.04)	−0.02 (0.03)	−0.06 (0.04)	0.11 ** (0.03)
Nonmetro	−0.02 (0.04)	0.03 (0.03)	−0.04 (0.04)	0.04 (0.03)

N = 1567. * = *p* < 0.05 ** = *p* < 0.01 *** = *p* < 0.001. ^1^ Average Marginal Effect. Average Variance Inflated Factor (VIF) = 1.45. F-test for model significance: F(57,1510) = 6.63 *p* value < 0.000.

**Table 4:** Weighted ordered proportional logistic regression for the relationship between financial resources, food security status, and days of poor mental health in the past month.

Odds Ratio (Linearized Std. Err)	Model 1 N = 1567	Model 2 N = 1567	Model 3 N = 1567	Model 4 N = 1567
Marginal food security	1.85 ** (0.37)	1.54 * (0.32)	1.52 * (0.31)	
Low food security	2.19 *** (0.38)	1.80 ** (0.32)	1.84 ** (0.35)	
Very low food security	3.74 *** (0.71)	2.66 *** (0.55)	2.68 *** (0.56)	
2019 annual income less than 130% FPL	1.09 (0.19)	1.02 (0.18)	0.98 (0.18)	1.02 (0.19)
Rent home		1.30 (0.19)	1.32 (0.19)	1.35 * (0.20)
Lost job in pandemic		1.70 (0.52)	1.70 (0.51)	1.81 (0.55)
Monthly income higher		0.79 (0.17)	0.79 (0.17)	0.87 (0.17)
Monthly income lower		1.65 ** (0.26)	1.58 ** (0.26)	1.75 *** (0.28)
Used savings		1.42 * (0.21)	1.44 * (0.21)	1.71 *** (0.25)
No savings available		1.18 (0.25)	1.15 (0.25)	1.52 (0.33)
Received stimulus check		1.27 (0.19)	1.28 (0.20)	1.28 (0.19)
Received charitable food		1.25 (0.17)	1.22 (0.17)	1.41 * (0.20)
SNAP			1.33 (0.26)	1.46 (0.29)
WIC			0.68 (0.14)	0.74 (0.16)
Unemployment Insurance			1.17 (0.21)	1.18 (0.22)
P-EBT			0.81 (0.14)	0.83 (0.15)
Age	0.98 *** (4.11 × 10^−3^)	0.98 *** (4.28 × 10^−3^)	0.98 *** (4.26 × 10^−3^)	0.98 *** (4.89 × 10^−3^)
Black	0.49 * (0.15)	0.51 * (0.16)	0.50 * (0.16)	0.54 * (0.17)
White	1.10 (0.23)	1.10 (0.24)	1.07 (0.24)	1.07 (0.23)
Hispanic	1.25 (0.34)	1.15 (0.31)	1.10 (0.29)	1.03 (0.28)
Presence of child	1.13 (0.17)	1.14 (0.17)	1.17 (0.17)	1.17 (0.18)
Less than a college degree	1.04 (0.14)	1.01 (0.13)	1.00 (0.13)	1.04 (0.13)
Midwest	0.68 (0.14)	0.66 * (0.14)	0.66 * (0.14)	0.64 * (0.13)
South	0.57 ** (0.10)	0.57 ** (0.11)	0.57 ** (0.11)	0.59 ** (0.11)
West	0.74 (0.15)	0.75 (0.16)	0.74 (0.16)	0.78 (0.17)
Nonmetro	0.91 (0.17)	0.92 (0.18)	0.93 (0.18)	0.95 (0.18)

* = *p* < 0.05 ** = *p* < 0.01 *** = *p* < 0.001. Average Variance Inflated Factor (VIF) for Model 1 = 1.53; Model 2 = 1.46; Model 3 = 1.51; Model 4 = 1.50. F-test for model significance: Model 1 [F(14,1553) = 11.34 *p* value < 0.000] Model 2 [F(22,1545) = 9.50 *p* value < 0.000] Model 3 [F(26,1541) = 8.22 *p* value < 0.000] Model 4 [F(19,1548) = 8.22 *p* value < 0.000].

## Data Availability

Due to the nature of this research, participants of this study did not agree for their data to be shared publicly, so supporting data is not available.
